# Syndecan-1 as a Biomarker for Cardiovascular Risk in Patients with Behçet’s Disease

**DOI:** 10.5152/ArchRheumatol.2025.11178

**Published:** 2025-12-01

**Authors:** Cengiz Kadıyoran, Pınar Diydem Yılmaz, Mevlüt Hakan Göktepe, Adem Küçük, Mustafa Oğul, Erkan Cüre, Faik Özdengül

**Affiliations:** 1 Department of Radiology, Necmettin Erbakan University Medical School, Konya, Türkiye; 2 Department of Internal Medicine, Necmettin Erbakan University Medical School, Konya, Türkiye; 3 Department of Rheumatology, Necmettin Erbakan University Medical School, Konya, Türkiye; 4 Department of Internal Medicine, Konya Numune Hospital, Konya, Türkiye; 5 Department of Internal Medicine, Gaziosmanpaşa Medicalpark Hospital, İstinye University, İstanbul, Türkiye; 6 Department of Physiology, Necmettin Erbakan University Medical School, Konya, Türkiye

**Keywords:** Atherosclerosis, Behçet di̇sease, cardiovascular risk, sydecan-1, cIMT

## Abstract

**Background/Aims:**

This study aimed to assess the potential of syndecan-1 levels as a biomarker for diagnosing cardiovascular diseases and atherosclerosis in patients with Behçet’s disease.

**Materials and Methods:**

After estimating the required sample size, 56 patients with Behçet’s disease and 56 age- and sex-matched controls were enrolled in the study. Carotid intima-media thickness (cIMT) was measured by a single radiologist using an appropriate technique. Syndecan-1 levels were determined with an Syndecan-1 human ELISA kit (CLOUD CLONE, USCN). The test system showed a correlation coefficient of 0.99 between expected and actual values, with a detection limit of around 0.01 ng/mL. The intra-assay coefficient of variation (CV) was under 10%, and the inter-assay CV was under 12%.

**Results:**

The data obtained from the study showed that syndecan-1 levels (*P *< .002) and cIMT measurements (*P* < .015) were significantly higher in patients with Behçet’s disease compared to the healthy control group. Additionally, correlation analysis revealed a significant negative relationship between syndecan-1 levels and cIMT. In a stepwise multiple regression analysis, a strong independent relationship was found between cIMT and age (beta [β] = 0.474, *P* < .001), syndecan-1 (β = 0.302, *P* = .007), and low-density lipoprotein (β = 0.219, *P* = .050).

**Conclusion:**

Syndecan-1 levels were higher in patients with Behçet’s disease compared to controls, and increased syndecan-1 may slow the progression of subclinical atherosclerosis. This study is a preliminary investigation. Further detailed studies are needed.

**Main Points**

- Syndecan-1 levels might be higher in patients with Behcet's disease.
- There is an inverse relationship between syndecan-1 and cIMT in patients with Behcet's disease.
- High syndecan-1 levels in patients with Behçet's disease may slow atherosclerosis progression.

## Introduction

Behçet’s disease is a chronic autoimmune disorder characterized by systemic inflammation, with an unclear etiology and a tendency for vascular involvement. Although it mainly affects the blood vessels, it can also involve multiple organ systems. Key clinical symptoms include eye problems, recurrent mouth ulcers, inflammation of both arteries and veins, arthritis, skin lesions, as well as gastrointestinal and neurological issues.^1,2^ In patients with Behçet’s disease, an overactive immune response causes widespread systemic inflammatory damage.^3^ The condition is more common in men than in women and generally follows a more severe course in male patients. It occurs more often in countries along the historic Silk Road, especially in the Middle East and East Asia. Its prevalence in Asian populations ranges from 110 to 120 per 100 000,^1,2^ while in Western populations, it is between 0.1 and 7.5 per 100 000.^4^ Türkiye has the highest prevalence worldwide, with regional variations from 20 to 421 per 100 000.^5^ Both genetic and environmental factors are believed to contribute to the development of Behçet’s disease.

In Behçet’s disease, cardiovascular involvement occurs with a frequency ranging from 7% to 46%.^6^ Carotid artery intima-media thickness (cIMT) is considered one of the risk factors for cardiovascular diseases.^7^ Although cIMT is associated with various cardiac risk factors, it also independently predicts future risk of myocardial infarction and stroke.^8^ Measuring cIMT has long been recognized as a reliable method for assessing the cumulative effects of early-stage atherosclerotic risk factors.^9^ Numerous studies demonstrate that cIMT values are significantly higher in patients with Behçet’s disease compared to healthy controls.^10,11^ In this context, cIMT measurement may serve as a valuable tool for the early diagnosis of atherosclerotic heart disease in individuals with Behçet’s disease.

Syndecan-1 (CD138), a type 1 transmembrane proteoglycan, belongs to the syndecan family, which includes 4 distinct members found in mammalian membranes. It consists of a core protein with an extracellular domain that is enriched with chondroitin- and heparan-sulfated glycosaminoglycan side chains.^12^ Initially identified in mammary epithelial cells, syndecan-1 participates in several vital biological processes, such as wound healing, cell adhesion, and motility.^13^ Low levels of syndecan-1 may contribute to increased inflammation, which can accelerate the development of atherosclerosis.^14^

A cross-sectional study suggested that elevated syndecan-1 levels may be related to disease activity in rheumatoid arthritis.^15^ In another study involving patients with rheumatoid arthritis, 6 weeks of disease-modifying antirheumatic drugs (DMARDs) treatment was linked to a decrease in serum syndecan-1 levels, independent of inflammatory effects.^16^ This study also showed that tumor necrosis factor alpha (TNF-α) inhibitors did not produce the same effect on syndecan-1 as DMARDs16. Research conducted on patients with psoriatic arthritis indicated that syndecan-1 might play a role in the mechanisms of the disease, such as leukocyte migration and retention in chronically inflamed synovium and angiogenesis.^17^ Additionally, syndecan-1 levels have been found to be high in patients with systemic lupus erythematosus.^18^ Contrary to these findings, another experimental study showed that syndecan-1 deficiency in rheumatoid arthritis is linked to increased bone erosion, cartilage destruction, and inflammation compared to wild-type arthritis and that syndecan-1 deficiency worsens disease severity in rheumatoid arthritis.^19^ Based on current data, more research is needed to clarify the role of syndecan-1 in the development and treatment of rheumatoid arthritis and other autoimmune diseases.

An experimental study investigating the role of syndecan-1 in cell adhesion and cytoskeleton reorganization examined the effects of cytochalasin D or colchicine.^20^ High-expressing transfectants were shown to bind and spread on immobilized thrombospondin or fibronectin, which are ligands for the heparan sulfate chains of proteoglycans. The study demonstrated that the core protein syndecan-1 promotes spreading by forming a multimolecular signaling complex at the cell surface that triggers cytoskeleton rearrangement. Cytochalasin D or colchicine inhibited this binding and spreading.^20^ In another study exploring whether the transient association of syndecan-1 with microfilaments plays a critical role in its biological functions, it was found that cytochalasin B affects syndecan-1 spreading and reorganization, whereas colchicine has no effect.^21^ Additionally, it has been reported that cyclosporine, by binding to cyclophilin A, may influence syndecan-1 levels, which play a significant role in reducing inflammation.^22^ According to the literature, the role of syndecan-1 in atherosclerosis, inflammation, and autoimmune diseases is confusing. These findings suggest that some drugs used in the treatment of Behçet’s disease may have either positive or negative effects on syndecan-1, and there may be a relationship between syndecan-1 levels and cardiovascular damage in Behçet’s disease, which has a complex etiology.

Behçet’s disease is marked by significant inflammation. However, atherosclerosis in these patients is less common than previously thought.^23^ Syndecan-1 levels in patients with Behçet’s disease and their potential link to atherosclerosis are not yet understood. Therefore, this study aimed to measure syndecan-1 levels in individuals diagnosed with Behçet’s disease and to examine its relationship with subclinical atherosclerosis.

## Methods

### Sample Size

Atherosclerosis is common in the general population.^24^ However, it is not as prevalent as thought in patients with Behçet’s disease.^23,25^ The estimated sample size for the study was calculated using the freely available G-Power software version 3.1.9.7 (Heinrich Heine University Düsseldorf; Düsseldorf, Germany). Considering a type 1 error of 5% (α = 0.05) and a type 2 error of 20% (β = 0.2; power = 80%), the minimum sample size was calculated to be 48 for each group and 96 in total. It was decided to include 15% more subjects than the calculated number in the study; therefore, 56 subjects were included in each group. Since most studies in the literature measuring cIMT thickness in patients with Behçet’s disease have involved 30-50 subjects,^23^ the sample size for the current research is within a reasonable range.

### Patient

The study group consisted of 56 patients over 18 who visited the hospital’s rheumatology clinic and were diagnosed with Behçet’s disease by a rheumatologist based on the International Working Group criteria. Pregnant women, patients under 18, and those with cardiovascular conditions (such as coronary artery disease, hypertension, heart valve disease, and heart failure), thyroid disorders, diabetes mellitus, hyperlipidemia, acute or chronic renal failure, neurological conditions (including cerebrovascular and demyelinating diseases), other rheumatological or autoimmune disorders, and malignancies were excluded from the study. Fifty-six individuals over the age of 18 who visited the hospital’s internal medicine and cardiology outpatient clinics with nonspecific complaints, did not have chronic diseases or take medications, agreed to participate in the study, had normal physical examinations and laboratory findings, and matched the study group in age and gender were included. The study was reviewed and approved by the Necmettin Erbakan University Meram Medical Faculty of Medicine Ethics Committee (Approval no.: 2022/3918; date: July 22, 2022). Written and verbal informed consent was obtained from all participants by the same author in accordance with the Declaration of Helsinki. All participants were referred to the Department of Radiology at Necmettin Erbakan University Medical Faculty. The medications and body mass index (BMI) of the Behçet’s group were analyzed alongside the basic clinical characteristics of the participants.

The levels of various peripheral blood parameters were measured in the participants, including hemogram, C-reactive protein (CRP), erythrocyte sedimentation rate, Homeostatic Model Assessment for Insulin Resistance (HOMA-IR), fasting plasma glucose (FPG), blood urea nitrogen, creatinine, uric acid, total cholesterol, triglycerides, high-density lipoprotein, low-density lipoprotein (LDL), aspartate aminotransferase, alanine aminotransferase, albumin, thyroid-stimulating hormone, fibrinogen, and syndecan-1. Additionally, cIMT measurements for the participants were obtained through radiological techniques.

### Carotid Artery Intima-Media Thickness Measurement

The cIMT for all participants in both the study and control groups was measured by a radiologist with expertise in vascular imaging. Measurements were performed in a blinded manner using a Siemens Acuson S3000 ultrasound system equipped with a 9L4 linear transducer (operating at 4.0-9.0 MHz). The carotid system was evaluated with the patient in a supine position, the neck slightly extended, and turned away from the side being examined. This assessment was conducted using B-mode, pulsed Doppler mode, and color Doppler mode. The intima-media thickness was measured as the distance between the first echogenic line, adjacent to the vascular lumen, and the second echogenic line. For standardization purposes, this measurement was performed 2 cm proximal to the bifurcation of the common carotid artery.^26^ Carotid intima-media thickness measurements were consistently obtained from plaque-free arterial segments and were conducted by a single radiologist to minimize operator-related variability.

### ELISA Measurement

The blood samples taken were centrifuged at 4000 rpm for 5 minutes, and the serums were stored at −80°C. Serum syndecan-1 levels were measured using a commercial human Syndecan-1 ELISA kit (CLOUD CLONE, USCN) following the manufacturer’s instructions. Absorbance was read at 450 nm with an ELISA reader. The test system showed a correlation coefficient of 0.99 between expected and actual values, with a detection limit of approximately 0.01 ng/mL. The intra-assay coefficient of variation (CV) was below 10%, and the inter-assay CV was under 12%. Serum syndecan-1 levels are reported in ng/mL, within a detection range of 1.56-100 ng/mL. The assay demonstrates high specificity for syndecan-1, with no significant cross-reactivity or interference from similar molecules.

### Statistical Analysis

SPSS version 26 (IBM SPSS Corp.; Armonk, NY, USA) was used for statistical tests. First, the homogeneity of the groups was checked using the Kolmogorov–Smirnov test. Homogeneous data were presented as mean ± SD, and non-homogeneous data were presented as median (range). The Pearson correlation test was employed for correlation analysis. Independent variables affecting the cIMT dependent variable were identified. A relationship was identified between cIMT value and syndecan-1, age, BMI, disease duration, LDL, glucose, and HOMA-IR. Stepwise regression analysis was used in multivariate regression analysis to identify independent variables affecting subclinical atherosclerosis. Stepwise regression analysis was conducted with cIMT as the dependent variable and Syndecan-1, age, BMI, disease duration, LDL, glucose, and HOMA-IR as independent variables. A *P*-value of <.05 was considered significant.

## Results

The age (40 ± 11.4 vs. 39.6 ± 9.6, *P* = .587) and gender distribution (30 [53.5%] vs. 28 [50.0%], *P* = .425) of the Behçet’s disease patients were comparable to those of the control group. Sociodemographic characteristics, including age, gender, organ involvement, and medication use among the Behçet’s patients, are detailed in Table 1.

**Figure 1.**
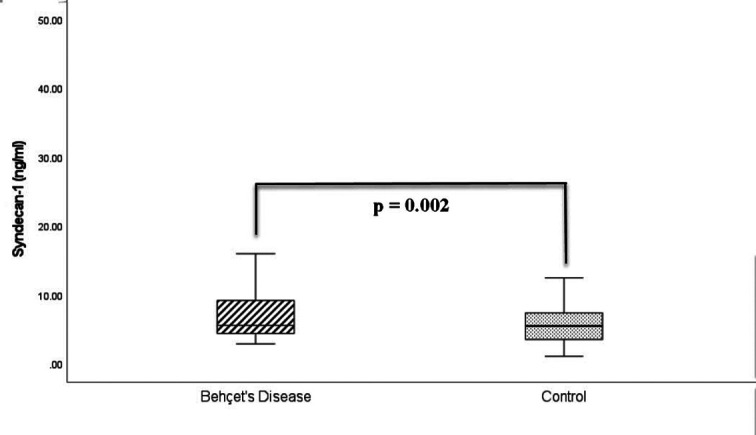
Serum syndecan-1 levels in both groups.

**Figure 2.**
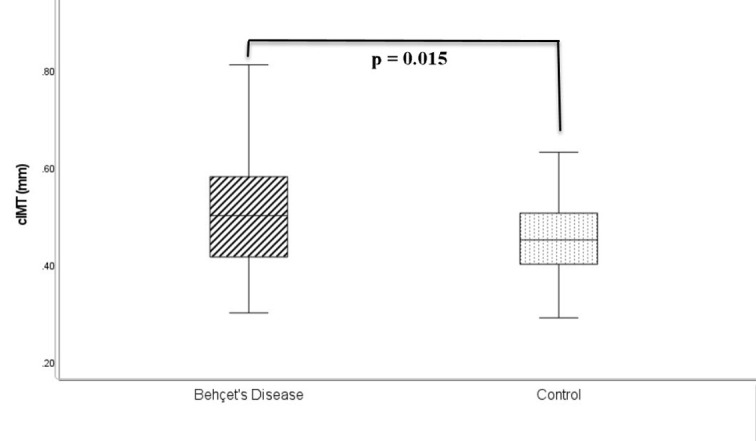
Carotid intima-media thickness values of both groups. cIMT, carotid intima-media thickness.

Syndecan-1 levels (9.6 ± 8.9 vs. 5.5 ± 3.2 ng/mL, *P* = .002), CRP levels (3.2 [0.3-41.7] vs. 1.2 [0.3-9.6] mg/dL, *P* < .001), and cIMT values (0.51 ± 0.1 vs. 0.46 ± 0.1 mm, *P* = .015) were significantly elevated in patients with Behçet’s disease compared to the control group. The complete biochemical profiles for both the Behçet’s disease patients and the control group are presented in Table 2. Syndecan-1 levels of both groups are given in Figure 1, and their cIMT measurements are shown in Figure 2.

**Figure 3.**
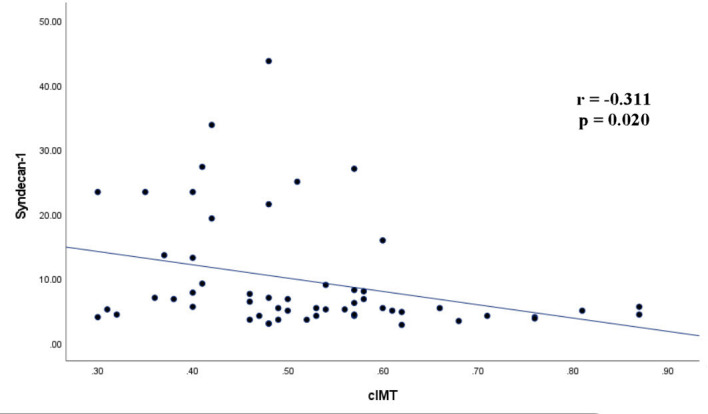
Carotid intima-media thickness and syndecan-1 correlation analysis results in Behçet’s patients. cIMT, carotid intima-media thickness.

Correlation analysis demonstrated a significant negative association between Syndecan-1 and cIMT (*r* = −0.311, *P* = .020). The correlation link between Syndecan-1 and cIMT is seen in Figure 3. No significant correlation was found between Syndecan-1 and disease activity and other parameters. Carotid intima-media thickness was positively correlated with age, BMI, FPG, HOMA-IR, and LDL levels. All the results of the correlation analysis are presented in Table 3.

In the current study, regression analysis was performed to identify which independent variables—syndecan-1, age, BMI, disease duration, LDL, glucose, and HOMA-IR—thought to be associated with subclinical atherosclerosis, have a substantial impact on the atherosclerotic process of patients in the study population. In a stepwise multiple regression analysis, a strongly independent relationship was found between cIMT and age (beta [β] = 0.474, *P* < .001), syndecan-1 (β = 0.302, *P* = .007), and LDL (β = 0.219, *P* = .050). No strong association was found between BMI, disease duration, glucose, and HOMA-IR values and the atherosclerotic process in this study population (all *P* > .05).

## Discussion

In this study, it was found that serum syndecan-1 levels in patients with Behçet’s disease were significantly higher compared to healthy controls. However, no strong correlation was observed between disease activity and circulating syndecan-1 levels. Correlation analysis showed a negative relationship between serum syndecan-1 levels and cIMT, while regression analysis suggested that lower syndecan-1 levels in patients with Behçet’s disease might be associated with subclinical atherosclerosis. Since this study was cross-sectional, further detailed research is needed.

Behçet’s disease is a chronic inflammatory condition with an etiology that remains largely unknown. The pathogenesis of Behçet’s disease is characterized by increased neutrophil infiltration, elevated levels of reactive oxygen species released from these cells, and the activation of monocytes and natural killer cells. Additionally, there is a rise in pro-inflammatory cytokines, including Interferon-gamma (IFN-γ), interleukin (IL)-12, IL-17, and IL-23, all of which play significant roles in its etiology.^27^ In Behçet’s disease, chronic inflammation is also associated with accelerated atherosclerosis and cardiac involvement. Carotid intima-media thickness serves as a reliable and cost-effective marker for subclinical atherosclerosis, effectively aiding in the early detection of endothelial dysfunction, which is a critical initial phase of the disease.^28^ In patients with Behçet’s disease, subclinical atherosclerosis may be present even at an early stage, and a plethora of studies have demonstrated that cIMT values in these individuals are significantly elevated compared to those of healthy controls.^10,11^ In this research, it was also found that the cIMT values in patients with Behçet’s disease were markedly elevated compared to those in healthy controls. On the other hand, studies are reporting that the frequency of subclinical atherosclerosis in Behçet’s disease is lower than in the general population. Studies examining subclinical atherosclerosis in patients with Behçet’s disease have been reported to involve small sample sizes of 30-50, which negatively affect the study results and may lead to a higher observed prevalence of atherosclerosis in these patients.^23^ It has been argued that the expected prevalence of atherosclerosis in patients with Behçet’s disease decreases as the size of the study population increases.^23^ Additionally, it has been suggested that using colchicine, immunosuppressive agents, and TNF-α blockers in patients with Behçet’s disease may reduce atherosclerosis in the carotid arteries, thereby maintaining carotid artery thickness despite increasing age.^25^ Since the increase in cIMT with age is clear in the general population, this claim by the authors may be accurate. Steroid therapy, commonly used by Behçet’s patients, can speed up the atherosclerotic process by raising both blood sugar and blood pressure.^29^ According to these results, the varying rates of subclinical atherosclerosis in patients with Behçet’s disease reported in the literature may be linked to the treatments the patients receive. There is a need for larger population studies that examine the effects of drug treatments on the atherosclerotic process in Behçet’s disease.

Glycocalyx proteins, primarily composed of hyaluronic acid and heparan sulfate, are located between the endothelial cell surface and the vascular environment, playing a crucial role in maintaining vascular integrity under physiological conditions. Syndecan-1 functions as a binding structure for hyaluronic acid and heparan sulfate within the endothelial transmembrane domain. It has been reported that syndecan-1 serves as a marker for the degradation of the endothelial glycocalyx.^30^ Syndecan-1 inhibits transendothelial leukocyte adhesion and transepithelial leukocyte migration.^31^ Under inflammatory conditions, elevated syndecan-1 levels may exert either pro-inflammatory or anti-inflammatory effects, potentially playing a significant role in the etiology of various diseases.^32^ It has been reported that syndecan-1 levels are elevated in inflammatory conditions such as diabetes, acute coronary syndrome, nephrotic syndrome, and sepsis.^33^ Zhou et al^33^ demonstrated that syndecan-1 levels are significantly higher in patients with septic shock compared to those with sepsis without shock and infections without sepsis.

The discovery of elevated syndecan-1 levels in acute coronary syndrome indicates a link between coronary artery disease and syndecan-1 levels.^34^ Two studies in children with Kawasaki disease also found higher serum syndecan-1 levels compared to healthy control children.^35,36^ These studies suggest that syndecan-1 levels are directly connected to cardiovascular involvement in Kawasaki patients. One study in children with Immunoglobulin A (IgA) vasculitis indicated that serum syndecan-1 levels might serve as a marker for vascular endothelial damage.^37^ Another study involving patients with antineutrophil cytoplasmic antibody (ANCA)-associated vasculitis suggests that serum syndecan-1 levels could predict disease activity and overall mortality.^38^ Overall, when these studies are considered, the increased syndecan-1 levels in Kawasaki vascular involvement, IgA vasculitis, and ANCA-associated vasculitis imply that syndecan-1 is linked to vascular participation in vasculitic diseases. However, these 4 studies were limited in number and cross-sectional. Some were also retrospective. This study was cross-sectional and involved a relatively small sample size. Therefore, more detailed research is necessary to determine whether syndecan-1 plays a role in vascular involvement in autoimmune diseases, including Behçet’s disease, and if so, to clarify the exact mechanism of this role.

The results of studies on syndecan-1 in the literature are contradictory. For example, 1 experimental study demonstrated that administration of recombinant syndecan-1 during myocardial infarction reduced histologically increased monocyte adhesion, endothelial glycocalyx damage, and cardiomyocyte damage.^39^ Conversely, an experimental study involving abdominal aortic clamping observed increased plasma syndecan-1 and pro-inflammatory cytokine levels, as well as increased cytokine levels in lung tissue, while syndecan-1 levels in lung tissue decreased.^40^ While it has been reported that increased syndecan-1 levels are linked to disease activity in patients with psoriatic arthritis and rheumatoid arthritis,^15-17^ a study also reports that syndecan-1 deficiency worsens the clinical condition in rheumatoid arthritis.^19^ In this study, there was no relationship between syndecan-1 and disease activity, CRP, and sedimentation. Miranda and colleagues identified a correlation between cIMT and syndecan-1 levels in patients with antiphospholipid syndrome.^41^ Conversely, in the current study, a negative correlation was observed between cIMT and syndecan-1 levels. It should be noted that the cross-sectional design of the studies and the number of subjects influence the results, as well as the potential effects of the drugs used in treatment on both syndecan-1 and cIMT, which may also impact the findings. It is known that drugs like colchicine, cyclosporine, and corticosteroids can influence serum syndecan-1 levels.^20,22,42^ Since immunosuppressive treatment is known to slow cIMT progression in patients with Behçet’s disease, it is likely that the treatment given to the Behçet’s patients in this study has influenced both cIMT and syndecan-1 values.^25^

In this study, serum syndecan-1 levels were measured. Tissue and serum syndecan-1 levels may vary, and their functions in inflammation might differ.^40^ On the other hand, Fukai et al^43^ claimed that intimal thickening resulted from carotid injury in syndecan-1 null mice and that syndecan-1 prevents intimal thickening. In contrast, Qiu et al^44^ asserted that syndecan-1 shedding in vulnerable plaques results in higher serum syndecan-1 levels. These findings indicate that syndecan-1 levels vary depending on different conditions and locations. Therefore, the role of syndecan-1 in atherosclerosis and its part in the etiology of autoimmune diseases should be clarified through large-scale, randomized, controlled population studies.

Circulating syndecan-1 levels were higher in patients with Behçet’s disease compared to healthy controls, regardless of disease activity. Syndecan-1 might be elevated in Behçet’s disease to help suppress inflammation. Additionally, increased circulating syndecan-1 levels could help slow the progression of subclinical atherosclerosis. This study did not examine how treatment agents affect syndecan-1 levels in patients with Behçet’s disease. Since this is the first study to investigate syndecan-1 levels in these patients, larger, randomized, controlled studies are needed.

### Limitation

First, the number of subjects included in the study is relatively small, and these patients have low disease activity indices. In this study, only circulating syndecan-1 levels were measured, while tissue levels may differ from cIMT measurements. This study conducted a cross-sectional assessment. Multi-center studies can produce safer and more comprehensive results. In patients with Behçet’s disease, circulating syndecan-1 levels can vary at different times. In this study, serum syndecan-1 samples from the subjects were only analyzed once. In this study, only serum syndecan-1 levels were examined. Tissue syndecan-1 levels may differ from those in serum. The patients in this study were on medication. Treatments like immunosuppressive agents, TNF-α blockers, corticosteroids, and colchicine could influence the rate of atherosclerosis and syndecan-1 levels. However, the impact of these treatments on the outcomes could not be assessed due to the small number of participants. The study results were assessed cross-sectionally by a single radiologist. Multiple measurements of cIMT or evaluations by multiple radiologists might influence the outcomes.^45^ This study is a pilot cross-sectional study with some limitations; more comprehensive studies are needed.

## Figures and Tables

**Table 1.** Sociodemographic Characteristics of the Patients with Behçet’s Disease

Parameters	BD (n = 56)
Disease duration (years) (mean ± SD)	12.8 ± 9.1
Disease activity median (range)	2.5 (1-6)
Treatment, n (%)	
Colchicine	49 (87.5)
Azathioprine	12 (21.4)
Steroid	9 (16.0)
TNF-α blocker	7 (12.5)
Cyclosporine	4 (7.1)
Methotrexate	1 (1.7)
Leflunomide	1 (1.7)
Involvement, n (%)	
Oral aphthae	52 (92.8)
Joint involvement, polyarthritis 9 (16.0), monoarthritis 8 (14.4), sacroiliitis 11 (19.6)	28 (50.0)
Pathergy test positivity	26 (46.4)
Genital ulcer	25 (44.6)
Eye involvement	21 (37.5)
Folliculitis	21 (37.5)
Vascular involvement	9 (16.0)
Thrombophlebitis	6 (10.7)
Erythema nodosum	3 (5.3)
CNS involvement, parenchymal	2 (3.5)
Kidney involvement, amyloidosis	1 (1.7)
GIS involvement (years)	0
Pulmonary aneurism	0

BD, Behçet’s disease; CNS, central nervous system; GIS, gastrointestinal system; TNF-α, tumor necrosis factor-alpha.

**Table 2.** Laboratory Results of the Patient and Control Groups

Parameters	BD (n = 56)	Control (n = 56)	*P*
Age (years)	40 ± 11.4	39.6 ± 9.6	.587
Gender (male), n (%)	30 (53.5)	28 (50.0)	.425
CRP (mg/dL)	3.2 (0.3-41.7)	1.2 (0.3-9.6)	<.001
ESR (mm/h)	8.0 (3.0-109.0)	6.5 (2.0-41.0)	.190
HOMA-IR	4.7 ± 4.4	2.8 ± 3.5	.013
BMI (kg/m^2^)	26.3 ± 4.9	26.9 ± 4.1	.513
FPG (mg/dL)	97.5 ± 25.1	90.9 ± 8.2	.069
BUN (mg/dL)	29.4 ± 8.2	25.3 ± 7.1	.006
Creatinine (mg/dL)	0.8 ± 0.1	0.8 ± 0.2	.457
Uric acid (mg/dL)	4.8 ± 1.1	4.8 ± 1.1	.845
Total cholesterol (mg/dL)	179.9 ± 38.6	192.1 ± 40.4	.105
Triglyceride (mg/dL)	160.0 ± 111.2	132.5 ± 87.0	.149
HDL (mg/dL)	44.7 ± 10.4	51.3 ± 12.1	.002
LDL (mg/dL)	103.0 ± 31.2	112.9 ± 35.6	.120
AST (U/L)	18.1 ± 9.5	17.8 ± 6.1	.828
ALT (U/L)	19.1 ± 12.5	18.5 ± 10.5	.786
Albumin (g/dL)	45.8 ± 2.7	47.1 ± 2.2	.008
TSH (µg/mL)	1.7 ± 1.1	1.9 ± 1.2	.377
Hb (g/dL)	14.3 ± 1.6	14.5 ± 1.9	.520
Fibrinogen (mg/dL)	316.6 ± 67.7	286.2 ± 57.3	.012

The results are presented as mean ± SD and median (range).

ALT, alanine aminotransferase; AST, aspartate aminotransferase; BD, Behçet’s disease; BMI, body mass index; BUN, blood urea nitrogen; CRP, C-reactive protein; ESR, erythrocyte sedimentation rate; FPG, fasting plasma glucose; Hb, hemoglobi; HDL, high-density lipoprotein; HOMA-IR, homeostasis model assessment of insulin resistance; LDL, low-density lipoprotein; TSH, thyroid-stimulating hormone.

**Table 3.** Correlation Analysis Results of Patients with Behçet’s Disease

	cIMT	
	*r*	*P*
Age	0.525	<.001
BMI	0.409	.002
FPG	0.307	.021
LDL	0.267	.047
HOMA-IR	0.290	.0030
Disease duration	0.461	<.001

BMI, body mass index; cIMT, carotid intima-media thickness; FPG, fasting plasma glucose; HOMA-IR, homeostasis model assessment of insulin resistance; LDL, low-density lipoprotein.

## Data Availability

The data that support the findings of this study are available on request from the corresponding author.
